# Sex differences in white matter integrity in youths with attention-deficit/hyperactivity disorder: a pilot study

**DOI:** 10.3389/fnins.2015.00232

**Published:** 2015-07-03

**Authors:** Jace B. King, Deborah Yurgelun-Todd, Amanda Stoeckel, Jennifer M. DiMuzio, Melissa P. Lopez-Larson

**Affiliations:** ^1^Department of Radiology, University of UtahSalt Lake City, UT, USA; ^2^Interdepartmental Program in Neuroscience, University of UtahSalt Lake City, UT, USA; ^3^Diagnostic Neuroimaging, University of UtahSalt Lake City, UT, USA; ^4^Department of Psychiatry, University of UtahSalt Lake City, UT, USA; ^5^University Neuropsychiatric Institute, University of UtahSalt Lake City, UT, USA

**Keywords:** ADHD, adolescents, DTI, fractional anisotropy, sex differences

## Abstract

Widespread disparities in white matter (WM) microstructure and organization have been found in adolescents with attention-deficit/hyperactivity disorder (ADHD); however, little is known about the role sex plays in these differences. The present diffusion tensor imaging (DTI) study performed whole-brain, tract-based, voxel-wise, and region of interest (ROI) analyses to investigate WM microstructure differences between ADHD and healthy control (HC) adolescents to examine the impact of sex on measures of fractional anisotropy (FA). Eighteen adolescents with ADHD and 24 HC were included in this study. All participants received a 64-direction DTI scan on a 3 Tesla Siemens scanner. FSL's TBSS was used to perform whole-brain, tract-based, voxel-wise analyses. Tracts demonstrating significant sex-by-diagnosis interactions were further evaluated using univariate analyses performed on mean FA data that were extracted from ROIs using the Johns Hopkins University WM tractography atlas. TBSS analyses between diagnostic groups revealed significantly increased FA in HC relative to ADHD in the bilateral superior longitudinal fasciculus (SLF), forceps major, left cingulum, and bilateral callosal regions. In addition, both TBSS and separate ROI analyses revealed significant sex-by-diagnosis interactions for the corticospinal tract (CST), inferior longitudinal fasciculus (ILF) and SLF. In the HC group, FA was increased in males relative to females for all analyses. In WM regions demonstrating a significant sex-by-diagnosis, FA was increased in females relative to males in the ADHD group. Our findings suggest that WM microstructure in several major WM tracts differs between males and females with ADHD. These differences in WM microstructure may account for some of the differences in ADHD subtypes and comorbidities seen between the sexes. Additional studies in ADHD, examining sex differences in phenotypic expression, treatment response and brain network trajectories are warranted.

## Introduction

Attention deficit/hyperactivity disorder (ADHD) is one of the most frequently diagnosed chronic neurodevelopmental disorders in children and adolescents with recent reports citing prevalence rates of up to 10% for United States (US) (Bloom et al., 2013) and non-US populations (Faraone et al., 2003). Furthermore, recent US health statistics report that male youths are three times more likely to be diagnosed with ADHD than females (Bloom et al., 2013); however, these differences in sex-related rates tend to converge in adult ADHD (Faraone et al., 2000) further supporting disparate clinical courses and neuropathologies between the sexes (Nussbaum, 2012). In studies comparing the phenotypic expression of ADHD symptoms in male and female youths, females are more likely to be diagnosed with the inattentive subtype of ADHD than males (Lahey et al., 1994; Biederman et al., 2002). In addition, as compared to males, females are more likely to exhibit internalizing symptoms and related comorbidities, such as generalized anxiety paired with the combined subtype of ADHD or separation anxiety disorder paired with the inattentive subtype (Levy et al., 2005).

Numerous structural and functional neuroimaging studies have identified widespread differences in youths with ADHD when compared to typically developing youths (Weyandt et al., 2013). For instance, brain regions such as the anterior cingulate cortex, prefrontal, temporal and parietal regions, insula, caudate, basal ganglia, corpus callosum, splenium, and cerebellum have been associated with the pathophysiology of ADHD (Silk et al., 2008; Qiu et al., 2009; Cao et al., 2010; Pastura et al., 2011; Lopez-Larson et al., 2012; Bledsoe et al., 2013). From a brain network perspective, fronto-striatal-cerebellar, default-mode, and attentional networks have been consistently reported as being atypical in ADHD (Konrad et al., 2006; Uddin et al., 2008; Cubillo et al., 2010). White matter (WM) volumes have also been reported to differ between youths with ADHD and healthy controls (HC). For example, Castellanos et al. (2002) reported a decrease in overall WM volume in ADHD when compared to HC.

To date, there have been no reports detailing sex-related differences in WM microstructure during development in youths with ADHD as compared to HC. However, studies of WM development in typically developing children and adolescents have identified significant sex-by-age interactions in WM volumes with males demonstrating a sharper positive WM tract growth trajectory when compared to females (De Bellis et al., 2001; Lenroot et al., 2007). Furthermore, recent evidence suggests that maturation of WM microstructure in the association, projection, and commissural tracts may occur earlier in adolescent females compared to males (Asato et al., 2010).

Diffusion tensor imaging (DTI) is a magnetic resonance technique used to investigate WM microstructure. The microstructure and organization of WM tracts is often reported using fractional anisotropy (FA), which is a measure of the degree of anisotropic diffusion of water molecules in brain tissue, which can be affected by cellular structure. Areas of high fiber organization where axons are more heavily myelinated, such as the corpus callosum, tend to have higher FA values. This imaging technique has been applied to the study of ADHD where differences in FA values have been found in ADHD relative to HC, though the directionality of these variances have been inconsistent (Ashtari et al., 2005; Hamilton et al., 2008; Pavuluri et al., 2009; Silk et al., 2009; Nagel et al., 2011; Peterson et al., 2011; Tamm et al., 2012; Lawrence et al., 2013). Additionally, in typically developing adolescents, WM FA has been found to vary according to sex; however, studies on this topic have also produced conflicting results (Bava et al., 2011; Herting et al., 2012; Wang et al., 2012). For instance, Bava et al. reported increased FA in females when compared to males in the bilateral corticospinal tracts (CST) whereas Wang et al. found increased FA in males relative to females in bilateral CST and left superior longitudinal fasciculus (SLF) tracts. Given the paucity of studies examining WM integrity in HC and in youths with ADHD, further examination of WM integrity across genders is needed.

To our knowledge, this is the first study that attempts to identify sex differences in WM microstructure in youths with ADHD as compared to HC. Based on previous literature documenting differences in the clinical expression of ADHD between the sexes and recent studies that indicate that WM matures differently in boys and girls, we first sought to determine if significant differences in WM FA could be detected in HC compared to ADHD youths, and second, to examine whether or not microstructural differences in WM can be discerned between male and female HC and ADHD youths. We performed both whole-brain, tract-based, voxel-wise comparisons and region of interest (ROI) measurements of DTI FA data to determine if differences existed between diagnostic groups as well as if sex indeed impacts WM microstructure differently between male and female youths with ADHD as compared to HC. We hypothesized that sex differences in WM microstructure in youths with ADHD would differ from that of HC youths. More specifically, we predicted that these differences would manifest as increased FA in females with ADHD as compared to males due to evidence suggesting earlier WM tract maturation in females with ADHD. Furthermore, we examined associations between WM integrity and measures of clinical symptoms often associated with ADHD including inattention and impulsivity/hyperactivity.

## Materials and methods

### Study participants

The Institutional Review Board at the University of Utah approved this study. All subjects provided written informed assent prior to study participation. Parental consent was also acquired for all study participants. Recruitment efforts included the use of local advertisements and word of mouth. A total of 43 subjects, including 19 youths with ADHD (10 male; 12.68 ± 2.14 years old) and 24 HC youths (12 male; 14.42 ± 2.76 years old), were included in this analysis. Inclusion criteria for study participants were: male or female, 10–18 years of age, and of any race or ethnicity. A clinical and diagnostic semi-structured interview (Kiddie Schedule for Affective Disorders and Schizophrenia for School-Age Children-Present an Lifetime Episode (K-SADS-PL) (Kaufman et al., 1997) was conducted for each study participant by a board-certified child psychiatrist (MLL) or psychologist (AS). Furthermore, information regarding any past or present medical and neurological disorders as well as any familial history of psychiatric disorders was obtained from the parents of the study participants. Tanner staging was assessed by showing youths and parents pictures/descriptors of pubertal development and asking them to rate their current stage based on these pictures/descriptors on a scale from 1 to 5 (Tanner, 1962). Inclusion criteria for ADHD subjects included a DSM-IV-TR diagnosis of either the inattentive, hyperactive/impulsive or combined subtype of ADHD. Youths with ADHD were allowed to continue currently prescribed medications; however, participants were asked to refrain from taking stimulant medications at least 24 h prior to scanning. Healthy control participants had no current or past history of a DSM-IV-TR Axis I diagnosis. Exclusion criteria for both groups included: major sensorimotor handicaps, full scale IQ < 70, autism, schizophrenia, bipolar disorder, conduct disorder, anorexia nervosa or bulimia, drug or alcohol dependence, active neurological or medical disease, current pregnancy or lactation, metal fragments or implants, a history of claustrophobia, or any other MRI scan contraindications.

Internalizing and externalizing symptoms, common in children and adolescents with ADHD, were assessed using the Child Behavior Checklist (CBCL) (Achenbach and Rescoria, 2001). The ADHD Rating Scale (ARS) was used to assess impulsivity/hyperactivity and inattention (Dupaul, 1998). Full scale intelligence quotient (FSIQ) was assessed using the Wechsler Abbreviated Scale of Intelligence (Wechsler, 1999) and the DSM-IV-TR Global Assessment of Functioning (GAF) (American Psychiatric Association, 2000) was used to assess global functioning.

### DTI data acquisition

Acquisition of imaging data was performed at the Utah Center for Advanced Imaging Research (UCAIR) using a 3T Siemens Trio scanner. A DT-MRI GRAPPA sequence was obtained utilizing 64 directions, 2 diffusion weightings: *b* = 0, 1000 s/mm^2^, TE/TR = 88 ms/9 s; matrix = 128 × 128 on 256 × 256 FOV; 2 × 2 × 2 isotropic voxels, slice thickness = 2.0 mm with 0 gap, and 70 slices). Original images were transferred from the scanner in the DICOM format and coded. Participants' MRI scans were read by a neuroradiologist to rule out gross pathology.

#### DTI data processing and FA extraction

Processing of diffusion weighted imaging data was performed in FSL (www.fmrib.ox.ac.uk/fsl). In depth details and description of the DTI processing stream can be located on the FSL website. Briefly, images were first corrected for motion and eddy distortion; next, non-brain tissue was removed using BET (Smith, 2002); and finally, DTIFIT, part of the FDT toolbox (Behrens et al., 2003a,b; Smith et al., 2004, 2006; Woolrich et al., 2009), was used to generate individual FA maps. During each step of the preprocessing stream, the DTI data were visually inspected by a skilled technician (JBK) for significant distortion, motion and eddy artifact, as well as accurate skull removal prior to post-processing. The nonlinear registration tool FNIRT, which uses a b-spline representation of the registration warp field (Rueckert et al., 1999), was then used to align subjects' FA images into common 1 × 1 × 1 mm standard space (Andersson et al., 2007). As our study participants were adolescents, the image best representing the full study cohort was selected as the target image for the registration step in order to generate a more adolescent specific FA target image. This target image was then affine-aligned into MNI152 standard space after which the images for each subject were transformed merging the nonlinear transform to the target FA image with the affine transform from the target to MNI152 space. This transformation resulted in each subject's FA images being displayed in standard-space. These individual maps were then all merged into a single 4D FA image. A mean FA skeleton was subsequently derived from the mean FA image.

We performed whole-brain, tract-based, voxel-wise analyses, examining both diagnosis and sex-by-diagnosis interactions, using permutation testing in FSL using the Randomize tool set (Bullmore et al., 1999; Anderson and Robinson, 2001; Nichols and Holmes, 2002; Hayasaka and Nichols, 2003). The statistical model for permutation testing was designed to determine if there were significant diagnosis or sex-by-diagnosis effects while controlling for age. Within the Randomize program, we chose to use the Threshold-Free Cluster Enhancement option to eliminate the need to estimate a cluster-forming threshold. Both diagnosis and sex-by-diagnosis analyses were performed using 5000 permutations. Test-statistical maps were produced for diagnosis and sex-by-diagnosis analyses and voxels with a *p* ≤ 0.01 were considered significant. Clusters meeting threshold significance in the sex-by-diagnosis interaction analysis were identified and FA values were extracted to assess the directionality of interaction effects.

In an effort to further evaluate our findings, WM tracts identified as significantly different between sexes and diagnoses were further analyzed by creating ROIs of each major tract. Mean FA data were then extracted from each of these tracts for *post-hoc* analyses. The Johns Hopkins University WM tractography atlas (Mori et al., 2005; Wakana et al., 2007) was used to create masks for the right CST, right inferior fronto-occipital fasciculus (IFOF), right inferior longitudinal fasciculus (ILF), right SLF, the right temporal portion of the superior longitudinal fasciculus (SLF-T), and the right uncinate fasciculus (UF), Thresholding was set at a minimum of 25 and maximum of 100 to obtain a 75% probability of the voxels in each mask being within the corresponding tract. Mean FA values were then extracted for each study participant from the region where the mean FA skeleton mask overlapped each regional mask. Therefore, we extracted mean FA data from the center of the entire WM tract in each region and used the mean FA data to perform diagnosis and sex-by-diagnosis interactions via general linear model analysis.

### Data analyses

Statistical analyses were carried out using SPSS software (version 20) for Mac OS X. Independent student's *t*-tests and chi-square tests were performed for demographic and clinical variables. Both whole-brain, tract-based, voxel-wise analyses, and univariate analyses for specified WM tracts were performed focusing on diagnostic group differences and sex-by-diagnosis interactions. Age was used as a covariate in all univariate analyses. Pearson's correlations were performed between significant FA values and clinical variables including externalizing and internalizing behaviors from the CBCL as well as symptoms of impulsivity/hyperactivity and inattention from the ARS.

## Results

### Demographic and clinical measures

Participants in the ADHD group were diagnosed with either ADHD combined subtype (52.6%) or inattentive subtype (47.4%). Of the 19 participants with ADHD, 16 had a familial history of ADHD. Familial history data was not available for one study participant due to adoption. Four of the ADHD participants (21.1%) had been diagnosed with one past or current comorbid diagnosis including a history of depression (*n* = 1), current oppositional defiant disorder (*n* = 2), and current depression (*n* = 1). Of the 19 ADHD study participants, 68.4% were currently taking stimulant medications at the time of the study; and of the 31.6% who were not, 50.0% of those individuals had a history of stimulant pharmacotherapy. Four ADHD participants (21.1%) were currently taking psychotropic medications other than stimulants [divalproex (*n* = 1), sertraline (*n* = 2), bupropion (*n* = 1)].

Diagnostic groups were matched for sex; however, age was found to be higher in HC compared to ADHD (HC: 14.42 ± 2.76; ADHD: 12.68 ± 2.14) (see Table 1). No significant differences were found between diagnostic groups for FSIQ, whereas GAF was significantly decreased in the ADHD group relative to HC (*p* < 0.001). Symptoms of impulsivity/hyperactivity and inattention as well as internalizing and externalizing symptoms, as assessed by the ARS and CBCL, respectively, were found to be significantly increased in ADHD when compared to sex matched HC (all, *p* < 0.001). Male and female participants in the ADHD groups were similar in age, Tanner stage, FSIQ, GAF, ARS impulsivity/hyperactivity, ARS inattention, and CBCL internalizing and externalizing *t*-scores. In addition, male and female participants in the ADHD group were similar on current and past exposure to stimulant medications.

Table 1**Clinical demographics with between-group and within-group differences**.

**Table:** 

	**HC (*n* = 24)**		**ADHD (*n* = 19)**		
	***n***	**%**	***n***	**%**	***X*^2^**
Sex (male)	12	50.00	10	52.60	Ns
	**Mean**	**STD**	**Mean**	**STD**	***p***
Age	14.42	2.76	12.68	2.14	0.03
Tanner stage	3.63	1.47	2.95	1.55	Ns
GAF	91.65	3.77	69.06	10.50	<0.001
FSIQ	113.59	8.40	107.53	11.49	Ns
ARS impulsivity/hyperactivity	0.22	0.52	4.68	3.13	<0.001
ARS inattention	0.13	0.46	6.53	2.78	<0.001
CBCL internalizing *t*-score	44.91	7.55	57.11	11.80	<0.001
CBCL externalizing *t*-score	41.13	6.15	60.00	11.56	<0.001

**Table:** 

	**HC**					**ADHD**				
	**Male (*n* = 12)**		**Female (*n* = 12)**		***p***	**Male (*n* = 10)**		**Female (*n* = 9)**		***p***
	**Mean**	**STD**	**Mean**	**STD**		**Mean**	**STD**	**Mean**	**STD**	
Age	14.25	3.22	14.58	2.35	ns	13.40	2.07	11.89	2.03	ns
Tanner stage	3.42	1.73	3.83	1.19	ns	3.10	1.73	2.78	1.39	ns
GAF	91.45	3.21	91.83	4.37	ns	66.00	10.32	71.78	10.46	ns
FSIQ	113.17	9.27	114.10	7.68	ns	103.43	14.88	111.13	6.53	ns
ARS impulsivity/hyperactivity	0.36	0.67	0.08	0.29	ns	4.80	3.43	4.56	2.96	ns
ARS inattention	0.27	0.65	0.00	0.00	ns	7.50	1.35	5.44	3.58	ns
CBCL internalizing *t*-score	44.00	7.89	45.75	7.47	ns	53.10	12.02	61.56	10.42	ns
CBCL externalizing *t*-score	43.09	5.54	39.33	6.34	ns	60.40	12.43	59.56	11.24	ns

*GAF, Global Assessment of Functioning; FSIQ, Full Scale Intelligence Quotient; ARS, ADHD Rating Scale; CBCL, Child Behavior Checklist*.

### Whole-brain, tract-based, voxel-wise, diagnostic group comparison

**Figure 1:**
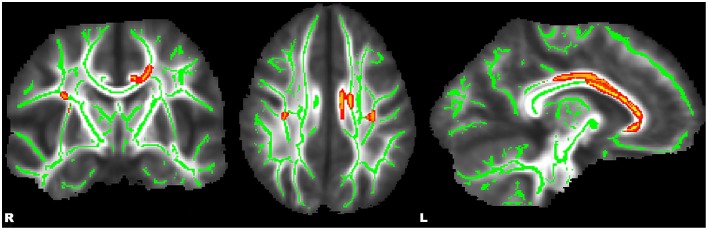
**Whole-brain, tract-based, voxel-wise comparison indicating regions with significantly increased fractional anisotropy (***p*** ≤ 0.01, corrected) in healthy control adolescents compared to adolescents with ADHD**. Significant regions were filled in using tbss_fill to improve visualization.

**Figure 2:**
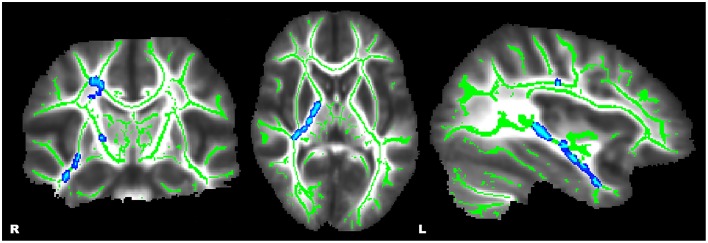
**Whole-brain, tract-based, voxel-wise comparison indicating regions with a significant sex-by-diagnosis interaction (***p*** ≤ 0.01, corrected)**. Significant regions were filled in using tbss_fill to improve visualization.

Whole-brain, tract-based, voxel-wise comparisons revealed significant main effects of diagnosis by way of increased FA in HC relative to ADHD in the bilateral SLF, forceps major (FMA), left cingulum (cingulate gyrus) (CGC), and bilateral callosal regions (*p* ≤ 0.01) (see Figure 1). Significant main effects of a sex-by-diagnosis interaction were evident in widespread regions located in right hemisphere regions of the CST, IFOF, ILF, SLF, SLF-T, and UF (*p* ≤ 0.001) (see Figure 2). Mean FA values were extracted from each of these clusters for *post-hoc* comparisons using univariate analyses. All five clusters indicated a significant sex-by-diagnosis interaction (*p* ≤ 0.001) with increased FA in males relative to females in the HC group, while FA was increased in females relative to males in the ADHD group.

### Mean FA measures

#### Diagnostic group effects on FA

For each study participant, mean FA values were extracted from masks within WM tracts identified in the whole-brain, tract-based, voxel-wise comparison. Univariate analyses, using diagnosis, sex, and sex-by-diagnosis as fixed factors and age as a covariate, were conducted on extracted FA values. Between diagnostic group analyses revealed significantly increased FA in HC relative to ADHD in the right [*F*_(1, 38)_ = 4.11, *p* = 0.05] SLF-T. No regions indicated significantly greater FA in ADHD when compared to HC.

#### Sex-by-diagnosis effects on FA

**Figure 3:**
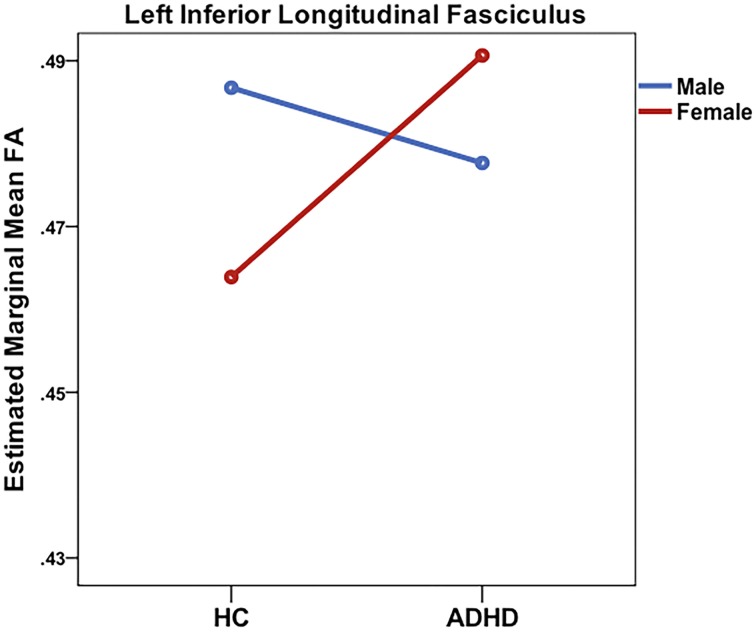
**Significant sex-by-diagnosis interactions in the right inferior longitudinal fasciculus from ROI analysis**.

Significant sex-by-diagnosis interaction effects were found for the right CST, ILF, SLF, and SLF-T (see Table 2 and Figure 3). In all instances where significant main effects of a sex-by-diagnosis interaction were found, FA was increased in males relative to females in the HC group, whereas in the ADHD group, FA was increased in females relative to males.

**Table 2:** **Raw fractional anisotropy values and results for the sex-by-diagnosis interactions between HC and ADHD for the region of interest analyses**.

**Region**	**HC (*n* = 24)**				**ADHD (*n* = 19)**				***F***	***P***
	**Male**		**Female**		**Male**		**Female**			
	**Mean**	**STD**	**Mean**	**STD**	**Mean**	**STD**	**Mean**	**STD**		
Right corticospinal tract	0.61	0.02	0.59	0.02	0.59	0.02	0.60	0.02	5.23	0.03
Right inferior fronto-occipital fasciculus	0.51	0.03	0.50	0.02	0.49	0.03	0.50	0.02	ns	ns
Right inferior longitudinal fasciculus	0.47	0.04	0.44	0.03	0.45	0.04	0.46	0.03	11.13	<0.01
Right superior longitudinal fasciculus	0.49	0.03	0.47	0.02	0.47	0.02	0.47	0.02	7.28	0.01
Right superior longitudinal fasciculus (temporal part)	0.54	0.03	0.52	0.02	0.50	0.03	0.52	0.02	10.05	<0.01
Right uncinate fasciculus	0.53	0.04	0.53	0.02	0.51	0.03	0.50	0.02	ns	ns

#### Clinical correlations with mean FA from ROI analysis

Correlations with hyperactivity/impulsivity and inattention scores as well as externalizing and internalizing symptom scores were performed on extracted FA values in tracts that had demonstrated significant sex-by-diagnosis interaction effects for the ADHD group, i.e., CST, ILF, and SLF/SLF-T. Correlations were also evaluated for each sex within the ADHD group. For the total ADHD group and in the male subgroup, no significant correlations were found with any clinical measure. However, females with ADHD demonstrated a significant positive correlation between mean FA in the right ILF and ARS impulsivity score (*r* = 0.72, *p* = 0.03).

## Discussion

This study found significantly increased FA in HC relative to ADHD in the bilateral SLF, FMA, left CGC, and bilateral callosal regions. Furthermore, sex-by-diagnosis interaction effects that appeared to be most prominent in right hemisphere CST, ILF, IFOF, SLF, SLF-T, and UF regions were also identified. In both the voxel-wise and univariate approach, increased FA was evident in males relative to females in the HC group, while FA was increased in females relative to males in the ADHD group in the identified regions. To our knowledge, this study offers the first evidence demonstrating that sex differences in WM microstructure are present in youths with ADHD.

Our finding of anomalous FA values in youths with ADHD when compared to HC agrees with current literature, but discordance exists within this area (Ashtari et al., 2005; Hamilton et al., 2008; Pavuluri et al., 2009; Silk et al., 2009; Nagel et al., 2011; Peterson et al., 2011; Tamm et al., 2012; Lawrence et al., 2013). For instance, in a recent study evaluating FA in youths with ADHD and age and sex matched controls, Peterson et al. (2011) reported increased FA in ADHD youths relative to HC in the right superior frontal and precentral gyri, the left lingual, parahippocampal, cingulate, and postcentral gyri, as well as the right posterior thalamic radiation. Similarly, in two other all male studies, increased FA in ADHD relative to HC was found in several major WM tracts throughout the brain including the UF, IFOF, anterior thalamic radiation (ATR), anterior corona radiata, and anterior forceps (Tamm et al., 2012) and in the left inferior temporal regions, left inferior frontal cortex/striatum, and right occipito-parietal cortex (Silk et al., 2009).

In contrast, Ashtari et al. (2005) reported decreased FA in children with ADHD (*n* = 18) when compared to age- and gender-matched HC (*n* = 15) in the left middle cerebellar peduncle, cerebellum, and parieto-occipital, and right cerebral peduncle, premotor, and striatal areas. Similarly, Nagel et al. (2011) found decreased FA in children with ADHD (*n* = 16) in frontoparietal, frontolimbic, cerebellar, corona radiata, and temporo-occipital regions when compared to HC (*n* = 20). Hamilton et al. (2008) also found decreased FA in youths with ADHD (*n* = 17), but in an all male sample, in the CST and SLF when compared to male HC (*n* = 16). Moreover, in a study comparing WM fiber tracts in youths with ADHD (*n* = 13), bipolar disorder (BPD) (*n* = 13), and HC (*n* = 15), Pavuluri et al. (2009) reported decreased FA in ADHD compared to HC the anterior corona radiata. Decreased FA was also reported in ADHD relative to both BPD and HC in the anterior limb of the internal capsule and superior region of the internal capsule. Given the significant sex-by-diagnosis interaction results of the current study, it may be reasonable to speculate that one reason for such discrepant FA findings in the literature on ADHD may be related to varying numbers of females or lack of female participants being reported on in the previously published studies. However, several additional factors may help account for the variable findings, including varying magnetic field strength, cohort age distribution, medication status, ADHD subtype, pubertal factors, and DTI data analysis methods.

The main sex-by-diagnosis interactions in FA between males and females with and without ADHD were found for the CST, ILF, SLF, and SLF-T, with males with ADHD having reduced FA and females with ADHD having increased FA as compared to HC. These major WM tracts have been associated with motor function, attention, impulsivity, and social skills, all of which are typically impaired in ADHD. The CST stretches between the primary motor cortex and the midbrain and is primarily involved in motor functions. The ILF connects the occipital and temporal lobes and reductions in FA in the ILF have been associated with thought disorder, social tasks, and cognitive dysfunction (Chanraud et al., 2010). The SLF extends posteriorly from the anterior cortex, through major lobules in both hemispheres and has been associated with verbal, attentional, and visual-spatial functions (Urger et al., 2015). The SLF-T is a tract that is an extension of the SLF and connects to the temporal lobe (Wakana et al., 2007). Given the role of these WM tracts in attention, impulsivity and motor functioning it is possible that the decrease in FA in the CST in males with ADHD may account for the motor abnormalities and the increased hyperactive subtypes seen in male youths (Hamilton et al., 2008). In addition, the decreased FA in the ILF and SLF may account for the disturbances in attention, impulsivity, and social dysfunction seen in males with ADHD. In support of our findings, Hamilton et al. also found decreased FA in the CST compared to HC in an all male sample of youths with ADHD and suggested that decreased FA in these regions may contribute to ADHD symptoms including inattention, impulsivity, and hyperactivity (Hamilton et al., 2008).

The higher FA values in females with ADHD in the CST, ILF, and SLF/SLF-T may account for the differences in clinical presentation (more inattentive and less motor/hyperactivity) and identified comorbidities (higher rates of anxiety) often seen in females with ADHD (Levy et al., 2005). Possible explanations for the current study's findings could be that the increase in FA in female youths with ADHD may be related to an earlier maturational development or a compensatory increase in FA for certain WM regions connecting the motor and attentional networks. This may in turn lead to reduced motor abnormalities in females while in other brain regions, such as the SLF and ILF, it may lead to increased inattention and impulsivity. Interestingly, in the current study, higher FA in the ILF in females with ADHD was also associated with higher ARS impulsivity scores. Furthermore, recent evidence suggests that maturation of WM microstructure in the association, projection, and commissural tracts may occur earlier in adolescent females compared to males (Asato et al., 2010). Given the scarcity of data detailing the structural and/or functional impact of sex on brain networks in youths with ADHD, additional studies with a larger sample size are needed to fully appreciate the current study's findings.

Our findings of sexual dimorphism in FA values in HC youths are consistent with some but not all of the existing research (Bava et al., 2011; Wang et al., 2012). For example, in a recent study that used tract-based voxel-wise comparisons to investigate the influence of age and sex on WM in 16 typically developing sex-matched adolescents, Wang and colleagues found increased FA in males relative to females in bilateral CST and left SLF tracts (2012). No regions were identified where FA was increased in females compared to males. The findings in Wang et al. are supportive of the current study's findings of reduced FA in typically developing females as compared to males. However, in a study that included 59 sex-matched healthy adolescents, Bava et al. reported increased FA in females relative to males in the right superior corona radiata and bilateral CST and no regions where FA was increased in males relative to females (2011). These discrepant findings between studies may be related to the age differences between studies, differing sample sizes, as well as data analysis methods.

Caution should be used when interpreting the results of this pilot study of sex effects on FA in ADHD. For example, the study was a cross-sectional study with modest sample sizes of male and female study participants in both the ADHD and HC groups. Although our sample size is modest, our male and female ADHD groups are similar on important variables such as FSIQ, ADHD severity measures, internalizing and externalizing symptoms and stimulant medication exposure. In addition, we obtained consistent findings using two different data analyses methods. Furthermore, though steps were taken to ensure consistent and accurate WM atlas registration, the Johns Hopkins University white matter tractography atlas was created using adult brains.

In summary, our pilot study examining the effects of sex on FA in major WM tracts in youths with ADHD as compared to HC demonstrated that WM microstructure might differ between males and females with ADHD. The differences in WM may account for the differences in ADHD subtypes and comorbidities seen between the sexes. Therefore, additional studies in ADHD examining sex differences in phenotypic expression, treatment response, and brain network trajectories may hold important clues to better our understanding of the pathophysiology of ADHD, which could then lead to improved diagnosis and treatments for both sexes.

## Author contributions

MLL and DYT were involved in all aspects of the research, including conception and design of the work, analysis, interpretation, and writing the manuscript. JBK was involved in the analysis, interpretation, and writing the manuscript. AS and JMD were involved in acquisition of the data.

## Funding

Research supported by NIMH: K23 MH087831 (MLL) and NIH: R01 DA020269 (DYT).

### Conflict of interest statement

The authors declare that the research was conducted in the absence of any commercial or financial relationships that could be construed as a potential conflict of interest.
